# Supplementary material for: Visualizing conformation transitions of the Lipid II flippase MurJ
Source: Nat Commun. 2019 Apr 15;10:1736. doi: 10.1038/s41467-019-09658-0 (PMC6465408; doi:10.1038/s41467-019-09658-0)
Supplement: Supplementary file 1 — Supplementary Information [file 41467_2019_9658_MOESM1_ESM.pdf]

Supplementary Information

Visualizing Conformation Transitions of the Lipid II Flippase MurJ

Kuk et al.

12 Supplementary figures

4 Supplementary tables

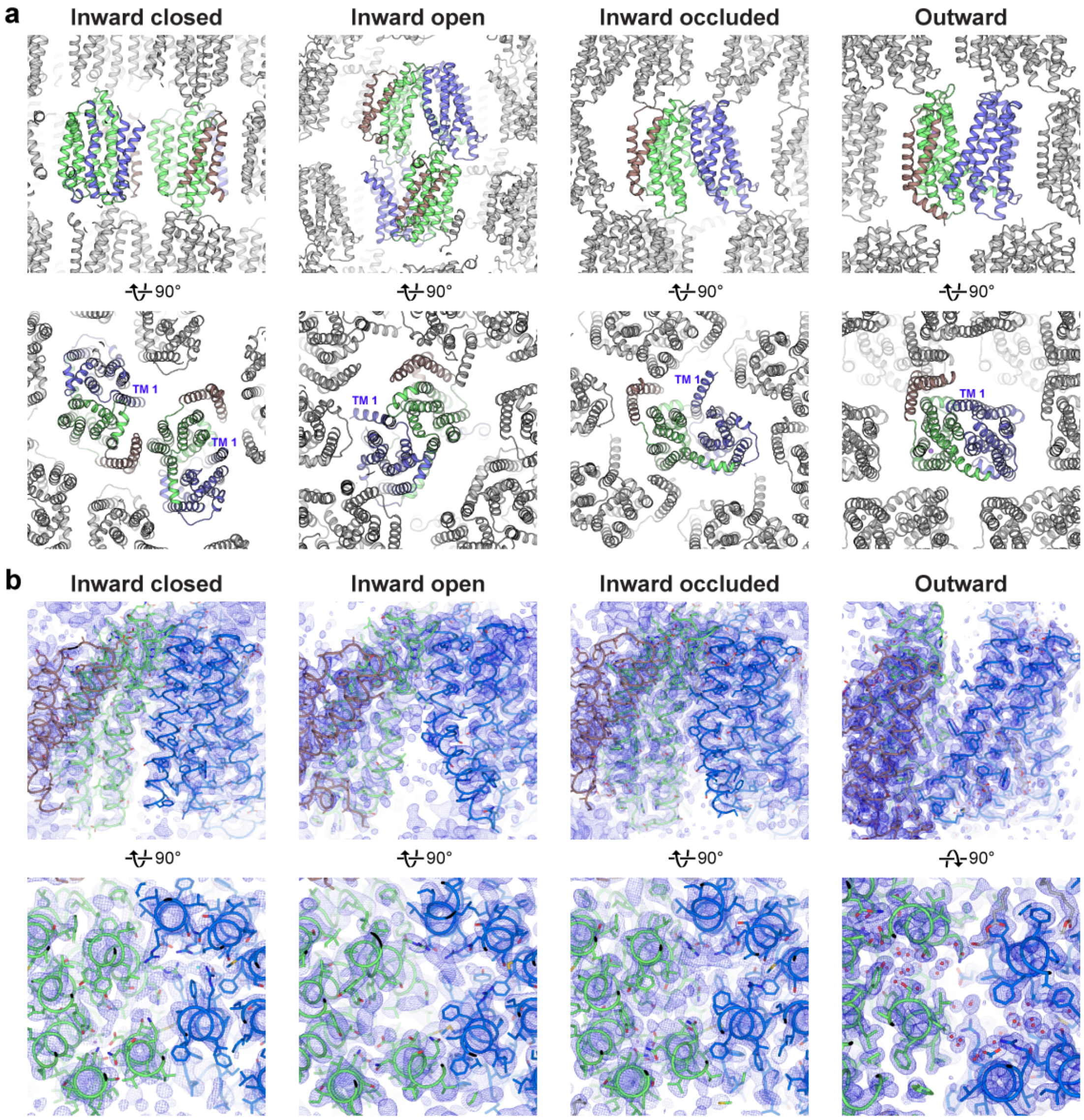

Supplementary Figure 1. Crystal packing and electron density in the MurJ$_{TA}$ structures. a. Crystals were obtained in lipidic cubic phase doped with Lipid II. Crystal contacts are mostly formed by end-to-end stacking, as well as lateral interactions mediated by the host lipid mono-olein. TM 1 was not involved in crystal packing and thus its bending is unlikely to be a crystallographic artifact. The asymmetric unit is colored, while symmetry mates are shown in gray. **b.** 2Fo − Fc composite omit electron density maps (contoured to 1 σ), omitting 5% of the model at a time. Resolutions are (from left) 3.2 Å, 3.0 Å, 2.6 Å, and 1.8 Å.

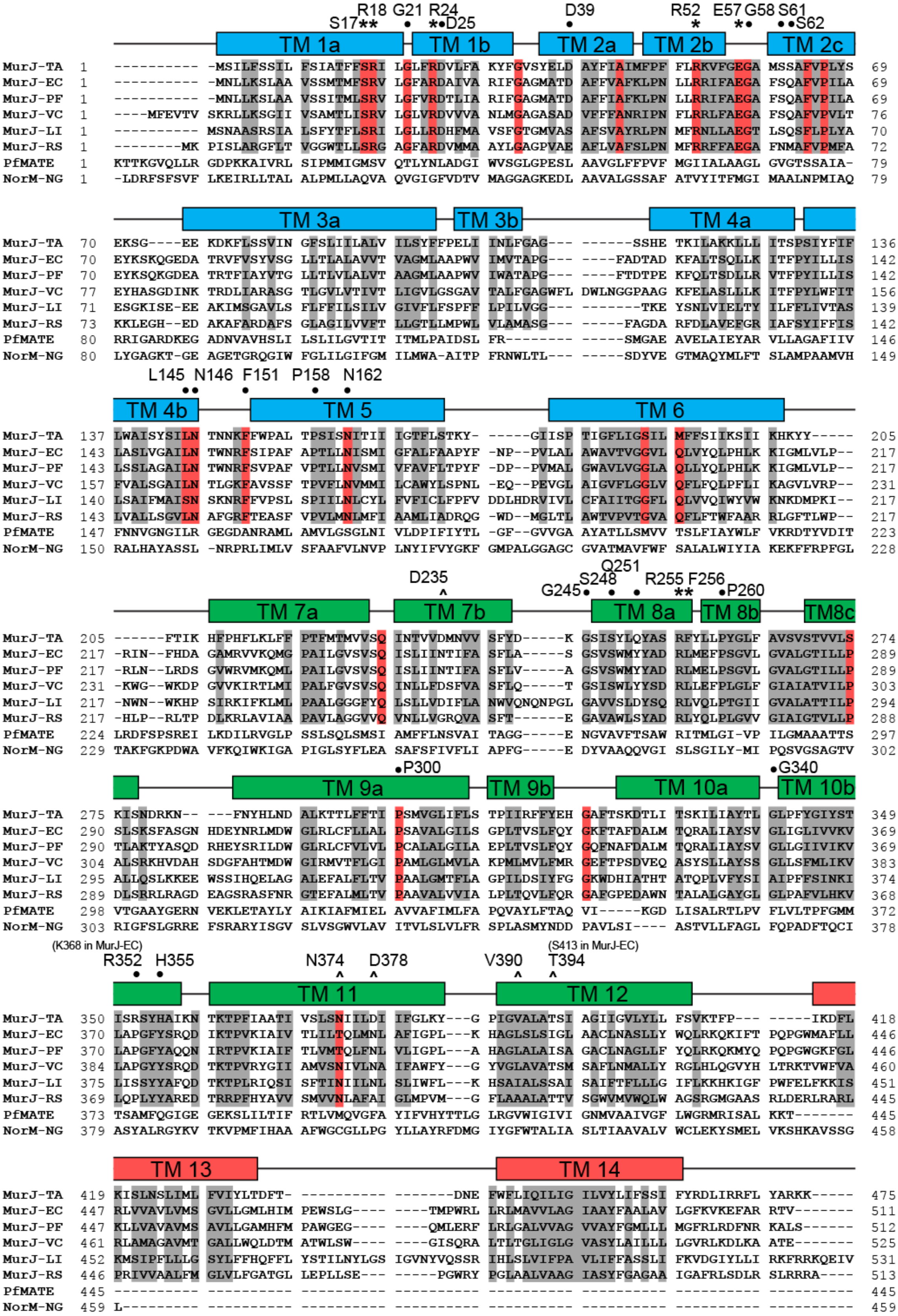

R18 G21 R24
S17 D25
D39
R52 E57 G58 S61
S62
TM 1a
TM 1b
TM 2a
TM 2b
TM 2c
MurJ-TA
MurJ-EC
MurJ-PF
MurJ-VC
MurJ-LI
MurJ-RS
PfMATE
NorM-NG
TM 3a
TM 3b
TM 4a
L145 N146 F151 P158 N162
TM 4b
TM 5
TM 6
D235
Q251
S248
R255 F256
G245
P260
TM 7a
TM 7b
TM 8a
TM 8b
TM8c
P300
G340
TM 9a
TM 9b
TM 10a
TM 10b
(K368 in MurJ-EC)
R352 H355
N374 D378
V390
(S413 in MurJ-EC)
T394
TM 11
TM 12
TM 13
TM 14

Supplementary Figure 2. Alignment of MurJ sequences. Positions shaded in red are identical in >90% of Gram-negative MurJ sequences in an alignment with 36 sequences, while those shaded gray are similar in >70% of the sequences. Asterisks (*) denote essential residues in MurJ_{TA} of which alanine mutants fail to complement *E. coli* MurJ, as determined previously [25]. Carets (^) denote residues participating in Na^+ coordination. Bullets (•) denote other residues discussed in this manuscript. MurJ_{TA} from *Thermosipho africanus* (UniProt ID: B7IE18) was aligned with 36 Gram-negative MurJ sequences from the UniRef50 library (no sequence is more than 50% identical to another). Drug exporters PfMATE and NorM-NG were also included in the alignment for contrast.

List of sequences shown: MurJ-TA, *Thermosipho africanus* (UniProt ID: B7IE18); MurJ-EC, *Escherichia coli* (P0AF16); MurJ-PF, *Pseudomonas fluorescens* (G8PWV6); MurJ-VC, *Vibrio cholerae* (O34238); MurJ-LI, *Leptospira interrogans* (A0A0C5X9A5); MurJ-RS, *Rhodobacter sphaeroides* (Q3J5H7); PfMATE (PDB ID: 3VVN); NorM-NG (4HUK).

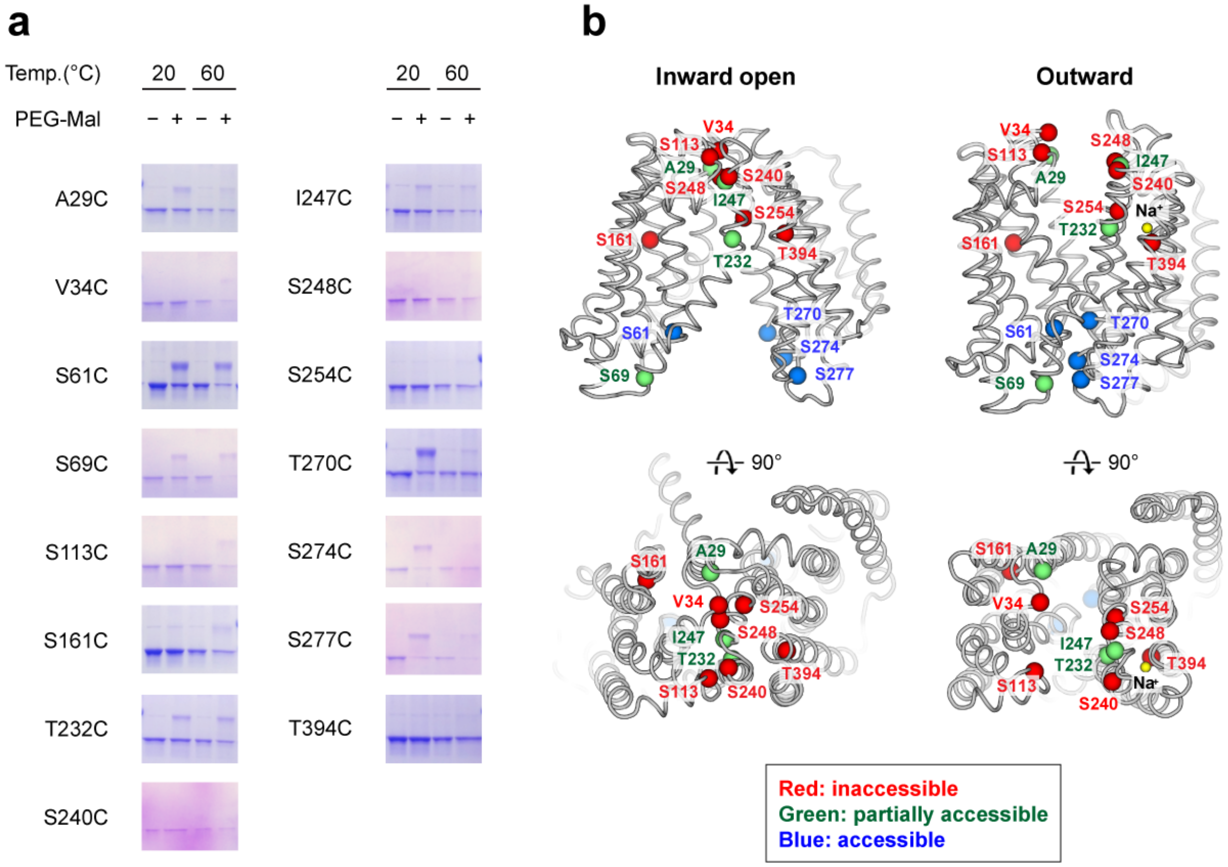

Supplementary Figure 3. MurJ$_{TA}$ assumes a predominantly inward-facing conformation in DDM micelles. a, Single cysteine mutants of MurJ$_{TA}$ (no endogenous cysteines) were expressed, purified, delipidated, and probed for accessibility in DDM micelles by a direct labeling strategy in potassium acetate (absence of sodium). Accessible cysteines would be labeled by PEG5000-maleimide (PEG-Mal), resulting in a mass shift up the gel. Experiments were performed at either 20°C or 60°C. Source data are provided as a Source Data File. **b,** Mapping of these cysteine positions (for the 20°C data) to the inward-open and outward-facing crystal structures of MurJ$_{TA}$. Positions were considered inaccessible if only the lower band was observed, and accessible if the top band was observed and of higher intensity than the lower band. Remaining positions with both bands visible were considered partially accessible.

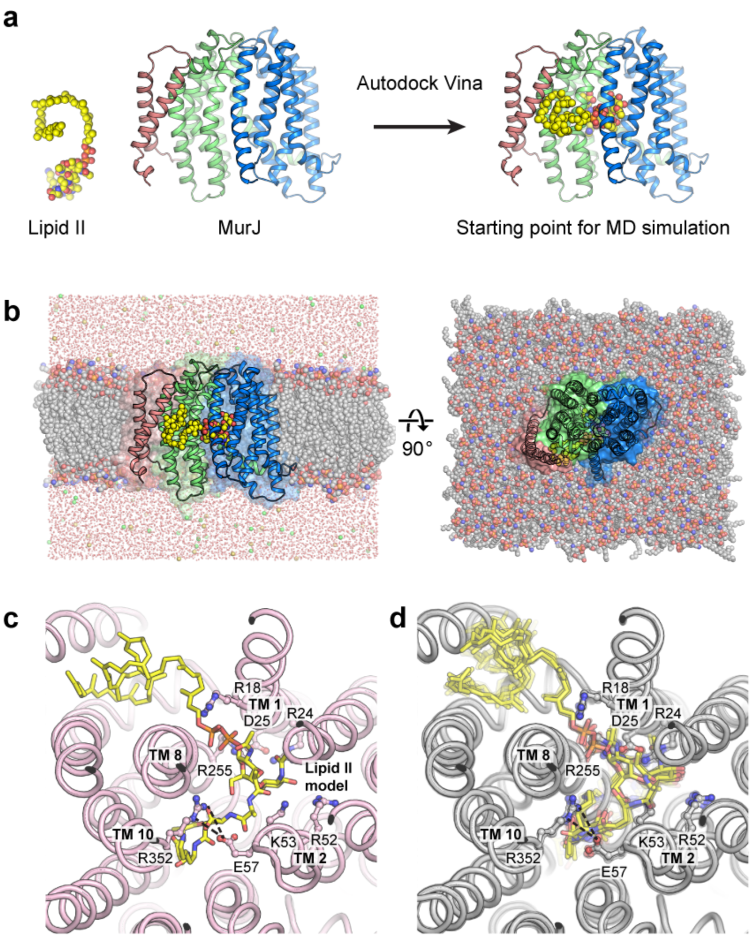

Supplementary Figure 4. Docking and MD simulation of Lipid II to the inward-occluded structure. a, Lipid II (yellow spheres) was docked to the inward-occluded structure by Autodock Vina. **b,** The docking result was used as a starting point for molecular dynamics simulation by NAMD in a hydrated and ionized (150 mM NaCl) POPE membrane bilayer system. The equilibrated system is shown, with lipids visualized as spheres and waters denoted by red dots. **c,** Cytoplasmic view of the docked complex (Lipid II shown as yellow sticks, charged residues in the cavity shown as gray sticks), showing how the Glu57-Arg352 thin gate could help occlude the substrate. **d,** Rotation of MurJ sidechain and Lipid II torsions in a molecular dynamics simulation starting from the docked model, visualizing the picosecond dynamics of the docked MurJ-Lipid II complex (snapshots at each 10 ps overlaid, total 100 ps). Surrounding lipids and waters are hidden for clarity.

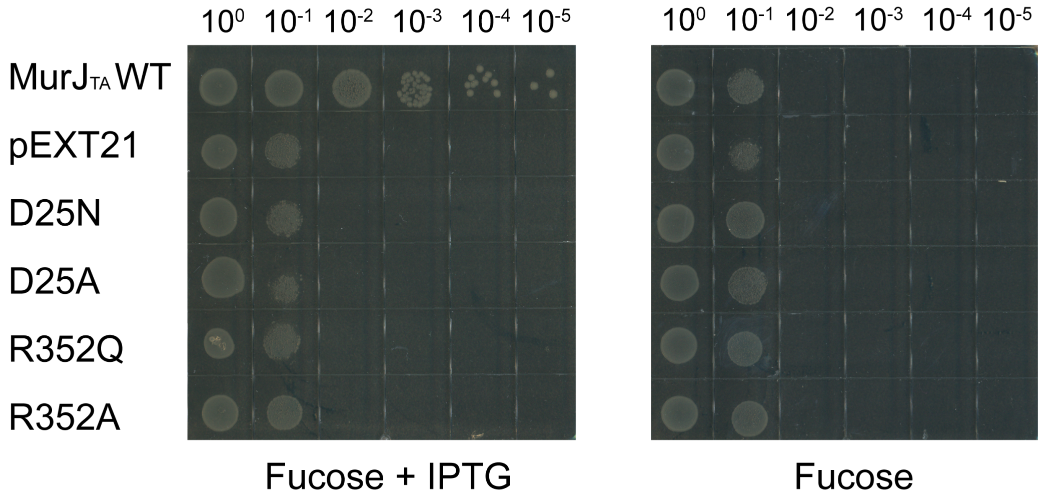

Supplementary Figure 5. Complementation assay of MurJ$_{TA}$ Asp25 and Arg352 mutants in *E. coli* NR1154. Cells transformed with plasmids encoding MurJ$_{TA}$ (wild-type or mutant) or without insert (pEXT21) were depleted of endogenous *E. coli* MurJ by serial dilution on plates containing the anti-inducer D-fucose. MurJ$_{TA}$ expression was induced by addition of IPTG. Data shown are representative of three biological replicates. Source data are provided as a Source Data File.

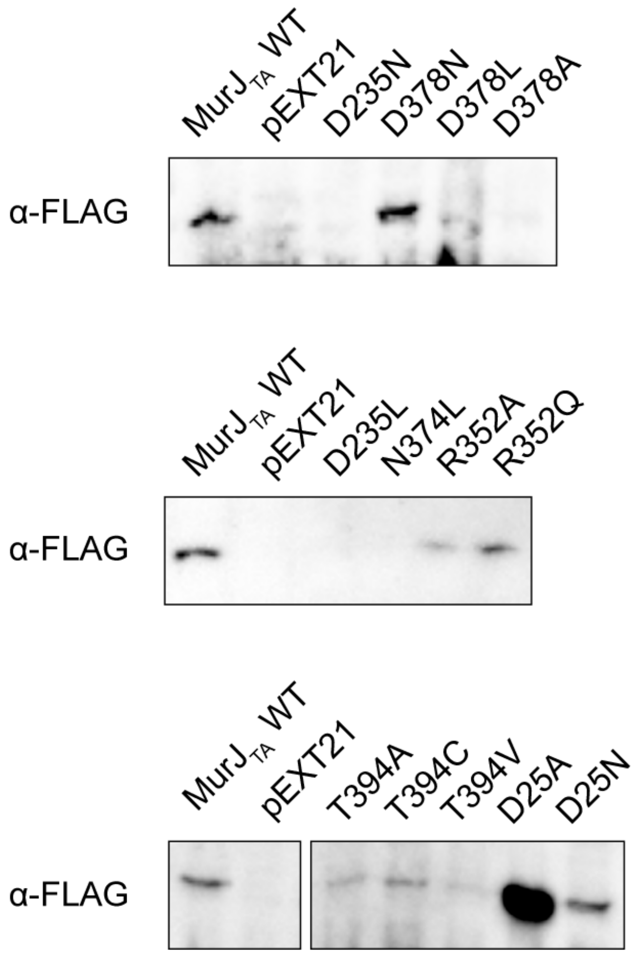

Supplementary Figure 6. Western blot analysis of MurJ$_{TA}$ mutants. MurJ$_{TA}$ proteins carrying a C-terminal FLAG tag were expressed by the addition of 0.1 mM IPTG in the presence of arabinose. Total membrane fractions were isolated by ultracentrifugation and probed by mouse anti-FLAG primary antibody and horseradish peroxidase-conjugated anti-mouse secondary antibody. Source data are provided as a Source Data File**.**

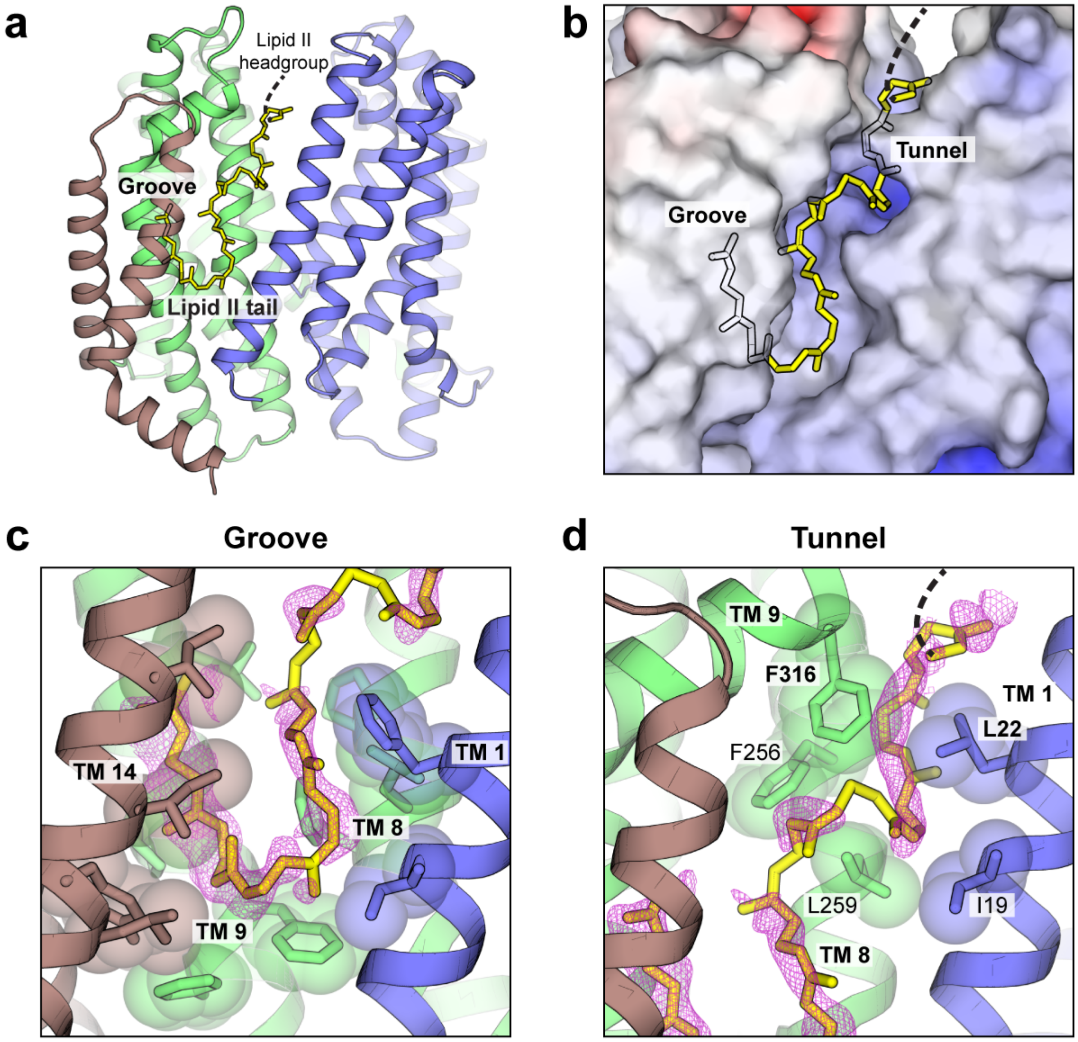

Supplementary Figure 7. Structural features of the putative lipid-binding site in the outward state. **a,** Putative lipid-binding site. Yellow sticks denote a model of the undecaprenyl tail of Lipid II, which was never used for structure refinement or map calculation. **b,** The lipid is restrained by the hydrophobic groove and a short tunnel near the periplasmic side. **c-d,** Unmodeled 2Fo − Fc electron density peaks (purple mesh, contoured to 0.7 σ) were observed in the hydrophobic groove (c), or in the tunnel (d) which is formed by hydrophobic residues Ile19, Leu22, Phe256, Leu259, and Phe316.

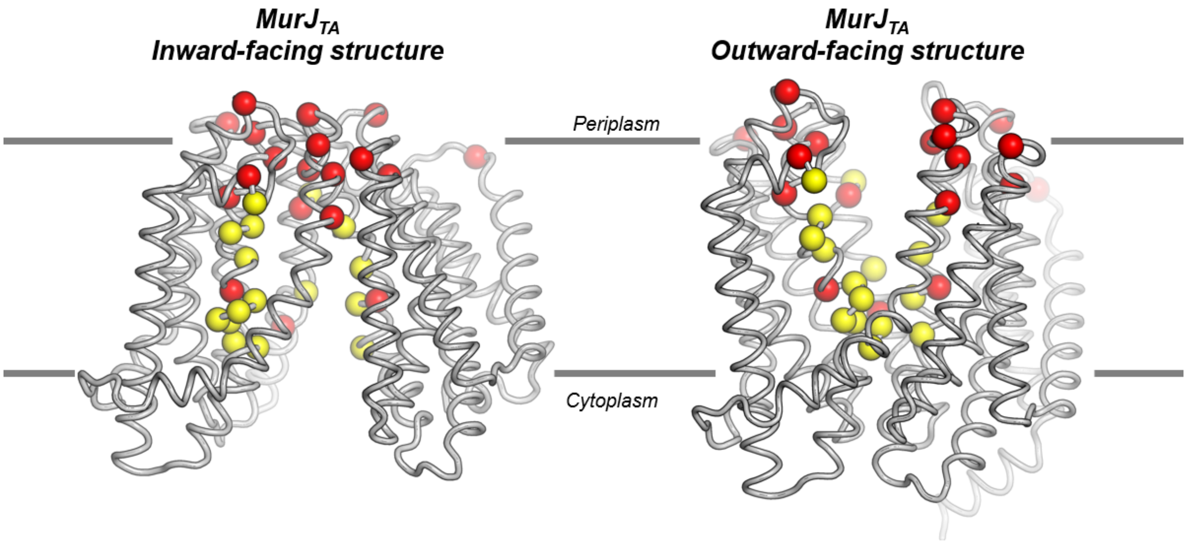

Supplementary Figure 8. Mapping of MurJ$_{EC}$ cysteine accessibility data (Butler *et al,* 2013) to MurJ$_{TA}$ structures. Positions accessible to the membrane impermeant reagent MTSES are shown as red spheres, while positions that are partially accessible are shown as yellow spheres. The structures of MurJ$_{TA}$ are consistent with the cysteine accessibility data suggesting a central cavity that is accessible to the periplasm (in the outward-facing conformation).

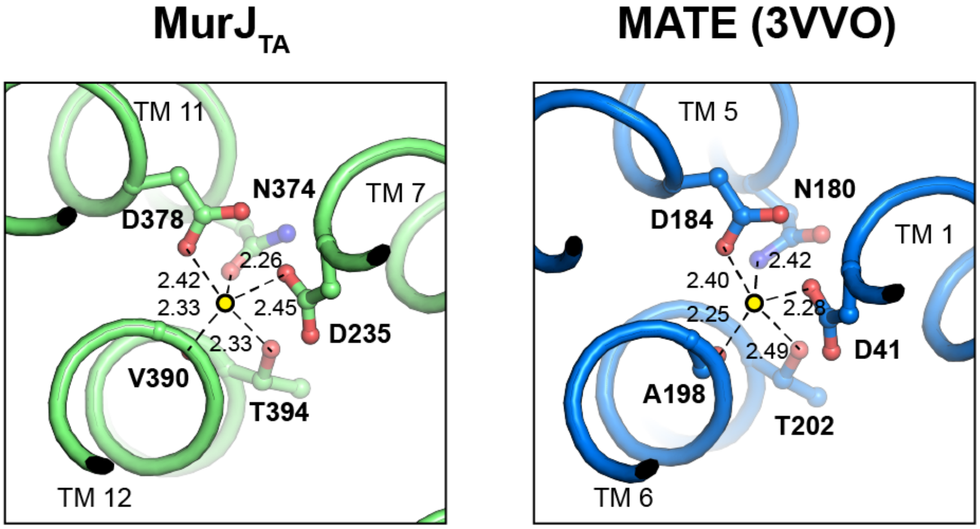

Supplementary Figure 9. The Na^+ site of $MurJ_{TA}$ is conserved with that of Na^+ coupled MATE transporters in the same superfamily. $MurJ_{TA}$ is shown on the left, while *Pyrococcus furiosus* MATE (PfMATE) is shown on the right (PDB ID: 3VVO, Tanaka *et al.,* 2013; Ficici *et al.,* 2018), both with a DNDXT composition where X is a backbone carbonyl oxygen.

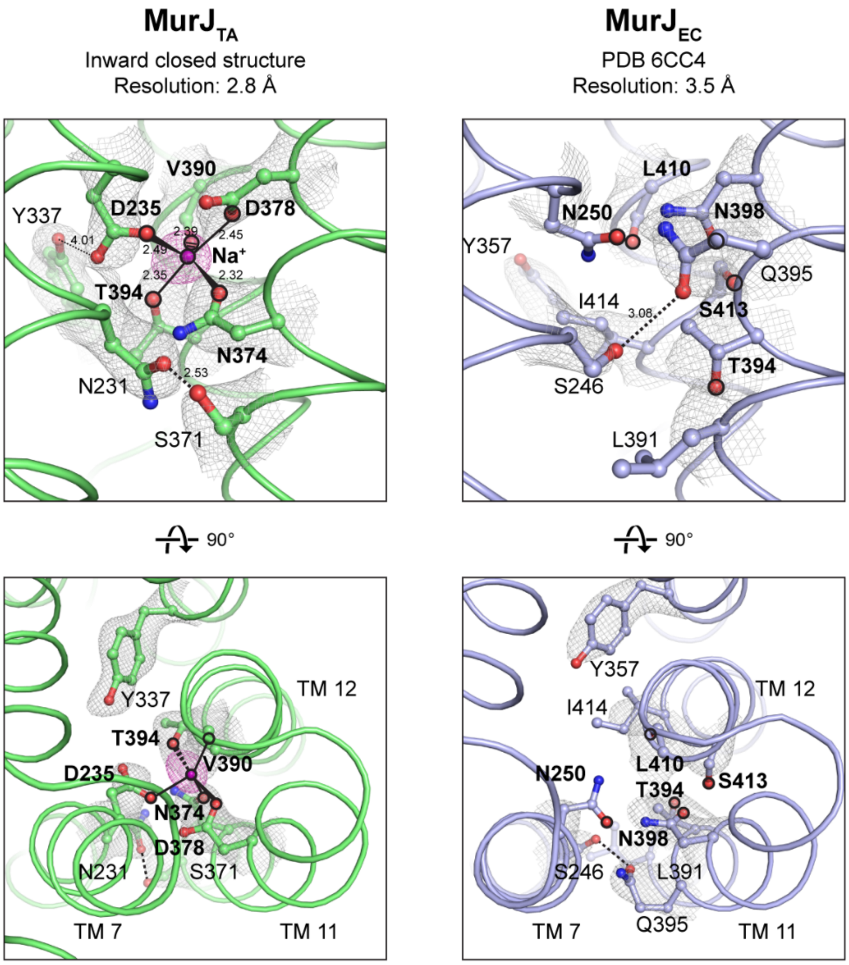

Supplementary Figure 10. The Na^+ site of $MurJ_{TA}$ is similar with that of $MurJ_{EC}$. $MurJ_{TA}$ is shown on the left, while $MurJ_{EC}$ is shown on the right (PDB ID: 6CC4, Zheng *et al.,* 2017). The putative sodium-coordinating oxygen atoms (black circles) are conserved.

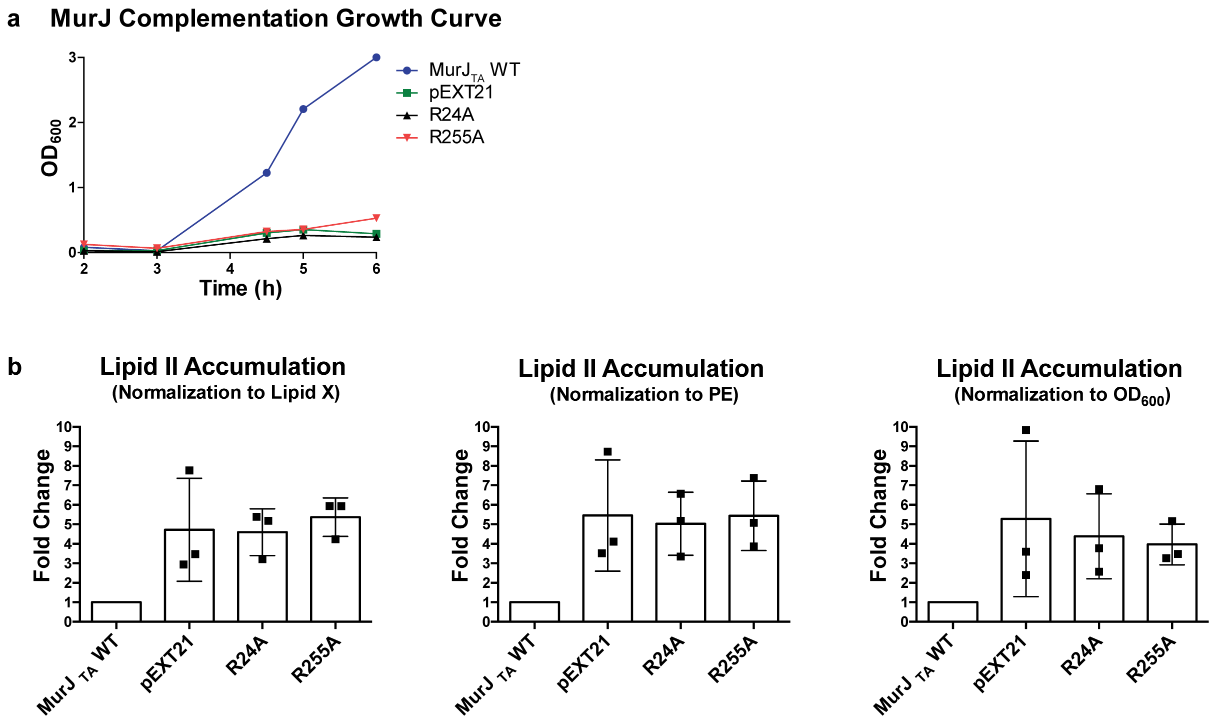

Supplementary Figure 11. Mass spectrometry assay of cellular Lipid II accumulation. a, Growth curve of *E. coli* MurJ-depletion strain NR1154 expressing $MurJ_{TA}$ wild-type (WT), mutants (R24A/R255A), or empty vector (pEXT21). Cultures were supplemented with D-fucose to deplete endogenous *E. coli* MurJ. At 3 hours post-inoculation, $MurJ_{TA}$ expression was induced with IPTG, and growth was monitored by optical density (OD_{600}). **b,** At 6 hours post-inoculation, cells were harvested and cellular Lipid II levels were analyzed by mass spectrometry. Cellular Lipid II levels were normalized against either Lipid X, phosphatidylethanolamine (PE), or cell density (OD_{600}). Lipid X is an intermediate in lipopolysaccharide biosynthesis pathway that is separate from the peptidoglycan synthesis pathway, but has similar chemical properties as Lipid II. Normalized Lipid II level of each replicate was divided by that of the wild-type to obtain the fold change. Data are mean ± s.d., n=3 biological replicates. Source data are provided as a Source Data File.

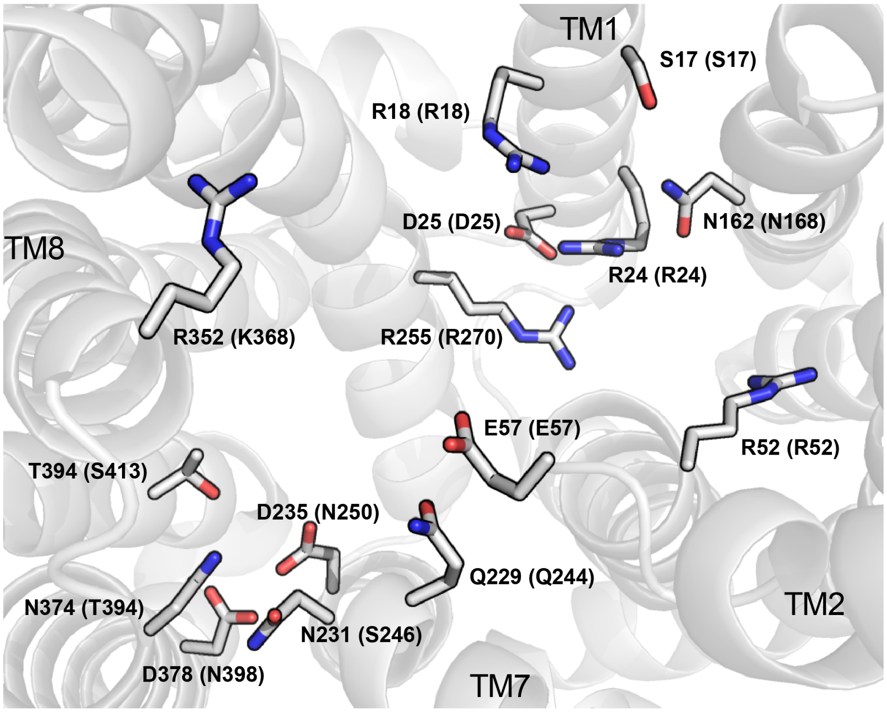

Supplementary Figure 12. Charged and polar residues in the central cavity of MurJ$_{TA}$ that were subjected to mutagenesis studies. Equivalent position in *E.coli* is in listed in parenthesis.

Supplementary Table 1. Data collection and refinement statistics (molecular replacement)

	**Inward closed**[a] **(6NC6)**	**Inward open**[b] **(6NC7)**	**Inward occluded**[c] **(6NC8)**	**Outward**[d] **(6NC9)**
**Data collection**				
Space group	$P\ 2\ 2\ 2_1$	C 2	$P\ 2\ 2_1\ 2_1$	C 2
Cell dimensions				
a, b, c (Å)	71.1, 101.8, 158.5	129.7, 105.5, 111.5	68.4, 80.3, 100.7	128.6, 57.4, 86.4
α, β, γ (°)	90, 90, 90	90, 125.3, 90	90, 90, 90	90, 100.7, 90
Resolution (Å)	85.6–3.2 (3.3–3.2)[e]	74.7–3.0 (3.1–3.0)[e]	68.4–2.6 (2.7–2.6)[e]	84.9–1.8 (1.9–1.8)[e]
R_{pim}	0.15 (0.94)	0.10 (0.83)	0.11 (>1.0)	0.076 (0.71)
$I/\sigma(I)$	9.48 (1.14)	11.41 (1.00)	13.29 (1.13)	22.71 (1.09)
Completeness (%)	99.4 (99.3)	99.0 (98.0)	99.8 (99.7)	99.9 (99.6)
Redundancy	20.7 (21.3)	5.5 (5.1)	48.8 (38.3)	23.4 (23.5)
**Refinement**				
Resolution (Å)	85.6–3.2 (3.3–3.2)[e]	74.7–3.0 (3.1–3.0)[e]	68.4–2.6 (2.7–2.6)[e]	84.9–1.8 (1.9–1.8)[e]
No. reflections	17624 (1292)	20395 (1238)	17459 (1608)	51025 (3345)
R_{work} / R_{free} (%)	25.5 / 28.0	25.4 / 27.8	22.9 / 25.8	17.9 / 19.9
No. atoms				
Protein	7292	7241	3672	3775
Ions/monoolein/PEG	27	126	125	294
Water	0	0	0	156
B factors (Å^2)				
Protein	31.18	46.54	48.19	20.55
Ions/monoolein/PEG	32.77	45.10	58.48	48.24
Water	N/A	N/A	N/A	28.35
R.m.s. deviations				
Bond lengths (Å)	0.005	0.005	0.003	0.003
Bond angles (°)	1.02	0.67	0.62	0.66

[a] Merged from 3 crystals.
[b] Merged from 2 crystals.
[c] Merged from 7 crystals (2 collected at wavelength of 0.98 Å and 5 at 1.65 Å).
[d] Merged from 6 crystals.
[e] Values in parentheses are for highest-resolution shell.

Supplementary Table 2. Comparison of mutagenesis data for MurJ_{TA} and MurJ_{EC}

		MurJ TA	MurJ EC					
**Residues in TA**	**Residues in EC**	Kuk et al., 2017, and this study	Butler et al., 2013, 2014	Zheng et al., 2018			**Location**
S17	S17	A			L W	P T A	TM1
R18	R18	A	A C E K	P	C S L	G H	TM1
R24	R24	A	A C E K	G	L Q	P	TM1
D25	D25	A N	C	H Y	N	G V A E	TM1
R52	R52	A	A C E K	C	S L P	H	TM2
E57	E57	A	C		K	Q A V G D	TM2
N162	N168	A		K*	I	D H Y S T	TM5
Q229	Q244	A	C		L	E K P R H	TM7
N231	S246	A			C	A P T F Y	TM7
D235	N250	A N L	C	K		D Y H T I S	TM7
R255	R270	A	A C E K	P	C S L	H	TM8
R352	K368	A Q	C		E I	Q T K N	TM10
N374	T394	A L		R K		P S A M	TM11
D378	N398	N L A				D H Y T S I K	TM11
T394	S413	C V A			Y	A P T F C	TM12

*Nucleotide 504 C to G and to A mutations, although resulting in synonymous N168K mutation, one led to a fold change of 13 and the other 2. In this table, we choose 13 for categorization.

Table Legend:

Kuk *et al.*, 2017 and this study,	Butler *et al.*, 2013 2014	Zheng *et al.*, 2018
No complementation	No complementation	Mutation frequency fold change >5
Partial complementation	Complementation but sensitive to low osmolarity	Mutation frequency fold change >2.5
Complementation	Complementation	Mutation frequency fold change <2.5

Supplementary Table 3. Sodium Fo − Fc omit peak heights and coordination distances

**Structure**	**Na^+ Fo − Fc omit peak height**	**Na^+ coordination distance (in Ångstrom)**				
		Asp235 OD1	Asn374 OD1	Asp378 OD1	Val390 O	Thr394 OG1
Inward closed	7 sigma	2.49	2.32	2.45	2.39	2.35
Inward occluded	9 sigma	2.44	2.43	2.40	2.40	2.41
Outward	19 sigma	2.45	2.26	2.42	2.33	2.33

Supplementary Table 4. List of primers used in this study

Primer name	Sequence (5' to 3')
**Sodium site**	
MurJ_D235L_F	agccagatcaacactgtggtgctaatgaacgtggtttctttctac
MurJ_D235L_R	gtagaaagaaaccacgttcattagcaccacagtgttgatctggct
MurJ_D235N_F	cagatcaacactgtggtgaacatgaacgtggtttctt
MurJ_D235N_R	aagaaaccacgttcatgttcaccacagtgttgatctg
MurJ_D378A_F	gcctgagcaatatcattctggctattatctttggcctgaaata
MurJ_D378A_R	tatttcaggccaaagataatagccagaatgatattgctcaggc
MurJ_D378L_F	gttagcctgagcaatatcattctgctaattatctttggcctgaaatacggc
MurJ_D378L_R	gccgtatttcaggccaaagataattagcagaatgatattgctcaggctaac
MurJ_D378N_F	ttagcctgagcaatatcattctgaatattatctttggcctgaaatac
MurJ_D378N_R	gtatttcaggccaaagataatattcagaatgatattgctcaggctaa
MurJ_N374L_F	cgcgaccattgttagcctgagcctaatcattctggatattatctttgg
MurJ_N374L_R	ccaaagataatatccagaatgattaggctcaggctaacaatggtcgcg
MurJ_T394A_F	gttgcgctggcggccagcattgcgg
MurJ_T394A_R	ccgcaatgctggccgccagcgcaac
MurJ_T394C_F	cgttgcgctggcgtgcagcattgcgggc
MurJ_T394C_R	gcccgcaatgctgcacgccagcgcaacg
MurJ_T394V_F	cgttgcgctggcggtcagcattgcgggc
MurJ_T394V_R	gcccgcaatgctgaccgccagcgcaacg
**Other mutagenesis**	
MurJ_D25A_F	cctgggtctgtttcgtgctgtactgttcgcaaaat
MurJ_D25A_R	attttgcgaacagtacagcacgaaacagacccagg
MurJ_D25N_F	gtatcctgggtctgtttcgtaatgtactgttcgcaaaatac
MurJ_D25N_R	gtattttgcgaacagtacattacgaaacagacccaggatac
MurJ_R352A_F	gcatctatagcaccattagcgctagctatcatgccatcaaaa
MurJ_R352A_R	ttttgatggcatgatagctagcgctaatggtgctatagatgc
MurJ_R352Q_F	ggcatctatagcaccattagccagagctatcatgccatcaaaaata
MurJ_R352Q_R	tatttttgatggcatgatagctctggctaatggtgctatagatgcc
**Cysteine accessibility**	
MurJ_A29C_F	gtctgtttcgtgatgtactgttctgtaaatacttcggtgtctcttacga
MurJ_A29C_R	tcgtaagagacaccgaagtatttacagaacagtacatcacgaaacagac
MurJ_I247C_F	ctttctacgacaaaggttcctgctcttatctgcaatacgcgt
MurJ_I247C_R	acgcgtattgcagataagagcaggaacctttgtcgtagaaag
MurJ_S113C_F	cctgttcggtgctggttgttctcatgaaactaag
MurJ_S113C_R	cttagtttcatgagaacaaccagcaccgaacagg
MurJ_S61C_F	cggtgagggtgcaatgtgttctgcatttgtacctc
MurJ_S61C_R	gaggtacaaatgcagaacacattgcaccctcaccg
MurJ_S69C_F	ctgcatttgtacctctgtactgtgaaaaatccggtg
MurJ_S69C_R	caccggatttttcacagtacagaggtacaaatgcag
MurJ_S161C_F	ggctctgactccgtctatctgtaacattaccatcattattg
MurJ_S161C_R	caataatgatggtaatgttacagatagacggagtcagagcc

MurJ_S240C_F	gtggacatgaacgtggtttgtttctacgacaaaggttc
MurJ_S240C_R	gaacctttgtcgtagaaacaaaccacgttcatgtccac
MurJ_S248C_F	cgacaaaggttccatctgttatctgcaatacgcgt
MurJ_S248C_R	acgcgtattgcagataacagatggaacctttgtcg
MurJ_S254C_F	tcttatctgcaatacgcgtgccgtttctacctgc
MurJ_S254C_R	gcaggtagaaacggcacgcgtattgcagataaga
MurJ_S274C_F	tatccactgttgttctgtgcaaaatctccaacgatcg
MurJ_S274C_R	cgatcgttggagattttgcacagaacaacagtggata
MurJ_S277C_F	ctgttgttctgtccaaaatctgcaacgatcgtaaaaacttcaa
MurJ_S277C_R	ttgaagtttttacgatcgttgcagattttggacagaacaacag
MurJ_T232C_F	tggtcagccagatcaactgtgtggtggacatgaacg
MurJ_T232C_R	cgttcatgtccaccacacagttgatctggctgacca
MurJ_T270C_F	ctgtttgctgtatccgtatcctgtgttgttctgtccaaaatctc
MurJ_T270C_R	gagattttggacagaacaacacaggatacggatacagcaaacag
MurJ_V34C_F	gttcgcaaaatacttcggttgctcttacgagctggatgca
MurJ_V34C_R	tgcatccagctcgtaagagcaaccgaagtattttgcgaac
